# The number of involved regions by prostate adenocarcinoma predicts histopathology concordance between radical prostatectomy specimens and MRI/ultrasound-fusion targeted prostate biopsy

**DOI:** 10.3389/fonc.2024.1496479

**Published:** 2024-12-11

**Authors:** Igor Yusim, Elad Mazor, Einat Frumkin, Ben Hefer, Sveta Li, Victor Novack, Nicola J. Mabjeesh

**Affiliations:** ^1^ Department of Urology, Soroka University Medical Center, Faculty of Health Sciences, Ben-Gurion University of the Negev, Be’er-Sheva, Israel; ^2^ Soroka Clinical Research Center, Soroka University Medical Center, Faculty of Health Sciences, Ben-Gurion University of the Negev, Be’er-Sheva, Israel; ^3^ Division of Diagnostic and Interventional Radiology, Soroka University Medical Center, Faculty of Health Sciences, Ben-Gurion University of the Negev, Be’er-Sheva, Israel

**Keywords:** prostate cancer, MRI/ultrasound fusion PB, targeted biopsy, systematic biopsy, radical prostatectomy, concordance

## Abstract

**Introduction:**

The prostate biopsy (PB) results should be concordant with prostatectomy histopathology to avoid overestimating or underestimating the disease, leading to inappropriate or undertreatment of prostate cancer (PCa) patients. Since the introduction of multiparametric Magnetic Resonance Imaging (mpMRI) in the diagnostic pathway of PCa, most studies have shown that MRI/Ultrasound fusion-guided (MRI-fusion) PB improves concordance with histopathology of radical prostatectomy specimens. This study aimed to evaluate the improvement in concordance of prostatectomy specimens with PB histopathology obtained using the MRI-fusion approach compared with the 12-core TRUS-Bx and to identify the variables influencing this.

**Patients and methods:**

The study included 218 men who were diagnosed with PCa by PB and underwent radical prostatectomy between 2016 and 2023. The patients were grouped based on the biopsy method: 115 underwent TRUS-Bx, and 103 underwent MRI-fusion PB. The histopathological grading of these biopsy approaches was compared with that of radical prostatectomy specimens. Multivariate logistic regression analyses were conducted to evaluate the impact of various criteria on histopathological concordance.

**Results:**

In patients with unfavorable intermediate- and high-risk PCa, MRI-fusion PB showed significantly better concordance with prostatectomy histopathology than TRUS-Bx (73.1% vs. 42.9%, p = 0.018). MRI-fusion PB had a significantly lower downgrading of prostatectomy histopathology than TRUS-Bx in all grade categories. The number of cancer-involved regions of the prostate is an independent predictor for concordance (OR = 1.24, 95%CI = 1.04-1.52, p = 0.02) and downgrading (OR = 0.46, 95%CI = 0.24-0.83, p = 0.01).

**Conclusions:**

Using an MRI-fusion PB improves histopathological concordance in patients with unfavorable intermediate and high-risk PCa. It reduces the downgrading rate of prostatectomy histopathology compared with TRUS-Bx in all grade categories. The number of cancer-involved regions is an independent predictor of the concordance between biopsy and final histopathology after prostatectomy and post-prostatectomy histopathology downgrading. Our findings could assist in selecting PCa patients for AS and focal treatment based on the histopathology obtained from the MRI-fusion PB.

## Introduction

Histopathological findings from prostate biopsies (PB) are crucial for determining potentially curative treatment for patients with prostate cancer (PCa) (1, 2). Therefore, the biopsy Gleason score (GS) should ideally be concordant with prostatectomy histopathology to avoid overestimating or underestimating the disease, leading to inappropriate or undertreatment (3). Previous studies reported that underestimation of the GS was a prevalent problem in classic 12-cores TRUS-Bx, with a prevalence as high as 43% (4). An early published meta-analysis showed limited concordance between TRUS-Bx and prostatectomy histopathology, reaching values smaller than 60%, with GS upgrading up to 30% and downgrading in 10% of cases (5).

In the last decade, multiparametric MRI (mpMRI) has become an essential diagnostic tool for detecting PCa. MRI/ultrasound fusion prostate biopsy (MRI-fusion PB) allows targeted biopsy (TB) of an MRI-suspicious lesion (6, 7). MRI-fusion TB improves the detection of clinically significant (csPCa) and reduces the detection of clinically insignificant PCa (ciPCa) (8–10). Combining TB with systematic biopsy (SB) results in greater detection of csPCa than either of these methods alone (8, 10–12). Since the introduction of mpMRI in the diagnostic pathway of prostate cancer, most studies have shown that MRI-fusion TB achieves concordance with prostatectomy histopathology between 60% and 80% (13–16). In addition, some studies have concluded that MRI-fusion TB combined with SB significantly increased the concordance with prostatectomy histopathology and decreased the upgrading rates (14, 17 **).**

Therefore, this study aimed to evaluate the improvement in concordance of radical prostatectomy specimens with PB histopathology obtained using the MRI-fusion approach compared with the traditional 12-core TRUS-Bx and to identify the variables influencing this.

## Patients and methods

We identified 1704 men who underwent PB at our institution between 2016 and 2023.

There were two distinct periods, each characterized by different strategies and methods of performing prostate biopsy. The first group consists of patients who underwent classical TRUS-Bx from the beginning of 2016 to the end of 2019. Since 2019, our institute has implemented the transperineal MRI-fusion PB method. Therefore, the second group consists of patients who underwent this procedure from late 2019 to the end of 2023. For men in group 1 who underwent TRUS Bx, the indications included an elevated or rising PSA level or a suspicious digital rectal examination and a family history of prostate cancer. For men in group 2 who underwent MRI-fusion TB and SB, the indication was mpMRI-suspicious lesions with a Prostate Imaging Reporting and Data System version 2.1 (PI-RADS) score ≥ 3 (18). Four men (3.9%) with clinical suspicion of PCa and a PI-RADS ≤ 2 underwent transperineal saturation SB and were included in the second group (19).

In the first group, we adhered to strict eligibility criteria for selecting patients for AS, in accordance with the EAU PCa guidelines recommendations at that time: clinical stage cT1c or cT2a, PSA levels < 10 ng/mL, International Society of Urological Pathology Grade Groups (ISUP GG) 1, and only two cancer-positive biopsy cores with ≤ 50% tumor involvement in each core (20). Patients who did not meet these criteria were offered curative treatment despite having ISUP GG1. The second group, after revising the inclusion criteria for the AC protocol, included patients with favorable intermittent-risk prostate cancer.

Two hundred eighteen patients with newly diagnosed prostate cancer who underwent radical prostatectomy were included in the study. The patients were divided according to the biopsy method: 1) men who underwent the standard 12-core TRUS biopsy and 2) men who underwent MRI-fusion TB and SB.

The position of each biopsy site was recorded using “Biojet Target, Biopsy Planning, Tracking, and Registration software,” which linked to ISUP GG for each biopsy core. This enabled us to create group 3 hypothetically from the selected TB-only data from group 2.

To analyze the concordance of ISUP GG of prostatectomy specimens with histopathology of TRUS-Bx, MRI-fusion TB and SB, and TB alone, we classified our patients into three subgroups based on ISUP GG: low-risk PCa (ISUP GG1), favorable intermediate-risk PCa (ISUP GG2), and unfavorable intermediate and high-risk PCa (ISUP GG ≥ 3) (21, 22). The ciPCa is defined according to the EAU PCa guidelines as GG1 (1).

### Biopsy techniques

A classic 12-core TRUS-Bx was performed under local anesthesia with a periprostatic nerve block. Extra cores were taken in the hypoechoic lesions on the TRUS image (23).

The transperineal MRI-fusion PB was performed under general anesthesia using the BioJet Target Release 3.0 (January 3, 2017, Medical Targeting Technologies GmbH, Kanalweg 7, 21357 Barum, Germany) image fusion system. We used a modified transperineal MRI-fusion PB technique consisting of 2 to 4 TB cores, followed by 20 SB cores taken from the five prostate regions (anterior and posterior bilateral and apex) (24).

We grouped biopsy cores by the regions from which they were taken for histological examination and received the detailed pathologist’s report of where the tumor was found.

### Statistical analysis

T-tests and Wilcoxon rank tests were used to compare the patients’ characteristics. Pearson’s Chi-squared test and Fisher’s exact test were used to compare the differences in concordance, upgrading, and downgrading prostatectomy pathologies from various PB. Multivariate logistic regression analysis was used to identify variables predicting concordance, upgrading, or downgrading of PB histopathology at prostatectomy. The analysis models were adjusted for clinical stage, PI-RADS score, PSA level at biopsy, prostate volume (PV), maximum cancer core length (MCCL), type of biopsy (MRI-fusion PB vs TRUS-Bx), and number of cancer-involved regions. Statistical significance is considered at p < 0.05. Data were analyzed using R Studio version 4.1.2 (2021 The R Foundation for Statistical Computing).

## Results

**Table 1** displays patients’ general demographics and characteristics in the TRUS-Bx and MRI-fusion PB groups. No significant differences were found between the patients in these groups regarding age, clinical stage, MCCL, PSA, or PSA density. In addition, when classified by ISUP GG, the histopathology results of PB and prostatectomy specimens were not significantly different. However, patients in the MRI-fusion PB group had a larger PV than the TRUS-Bx group.

**Table 1:** Descriptive characteristics of patients with prostate cancer who underwent radical prostatectomy, diagnosed with TRUS-Bx or MRI-fusion targeted and systematic PB (MRI-TB + SB).

	TRUS-Bx, N = 115	MRI-TB+SB, N=103	p-value^1^
**Age, y.** mean ± SD	64.6 ± 5.8	65.3 ± 5.5	0.4
Clinical stage, n (%)			0.06
T1C	98 (85.2)	74 (71.9)	
T2A	3 (2.6)	2 (1.9)	
T2B	11 (9.6)	21 (20.4)	
T2C	3 (2.6)	6 (5.8)	
**PSA**, ng/m; mean ± SD	7.8 ± 4.3	8.0 ± 4.0	0.5
**Prostate volume,** ml; mean ± SD	36.9 ± 22.8	44.4 ± 21.3	**<0.001**
**PSA density,** ng/ml^2^; mean ± SD	0.28 ± 0.23	0.22 ± 0.15 (103)	0.051
**MCCL**, mm; mean ± SD	6.4 ± 3.9	6.9 ± 4.2	0.9
PI-RADS, n (%)			
2		4 (3.9)	
3		25 (24.3)	
4		48 (46.6)	
5		26 (25.2)	
Biopsy ISUP GG, n (%)			0.4
GG 1	52 (45.2)	40 (38.9)	
GG 2	28 (24.4)	37 (35.9)	
GG 3	17 (14.8)	13 (12.6)	
GG 4	16 (13.9)	10 (9.7)	
GG 5	2 (1.7)	3 (2.9)	
Radical Prostatectomy ISUP GG, n (%)			0.2
GG 1	33 (28.7)	20 (19.4)	
GG 2	50 (43.5)	49 (47.6)	
GG 3	21 (18.2)	19 (18.4)	
GG 4	8 (7.0)	7 (6.8)	
GG 5	3 (2.6)	8 (7.8)	

^1^Wilcoxon rank test, Fisher’s exact test, Pearson’s Chi-squared test. Bold values: statistically significant p-values (p <0.05).

MCCL, Maximum cancer core length; PI-RADS, Prostate Imaging Reporting and Data System score v2.1; ISUP GG, International Society of Urological Pathology Grade Groups.

**Figure 1:**
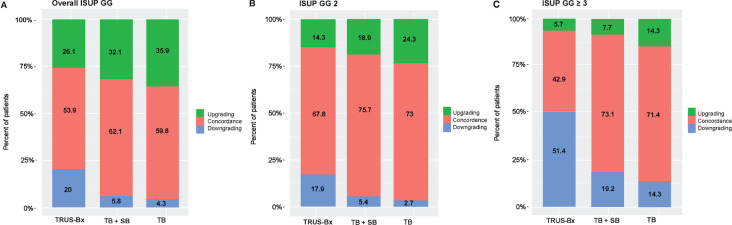
Rates of concordance, upgrading, and downgrading prostatectomy histopathology with MRI-fusion TB+SB, TB, and TRUS-Bx in **(A)** all ISUP GG, **(B)** ISUP GG 2, and **(C)** ISUP GG ≥ 3. TB + SB, MRI-fusion targeted + systematic PB; ISUP GG, International Society of Urological Pathology Grade Groups.

**Table 2** represents the correlation of ISUP GG of different biopsy techniques with prostatectomy histopathology. There were no significant differences in concordance between prostatectomy histopathology and the TRUS-Bx, MRI-fusion TB + SB, and TB groups for overall GG (53.9%, 62.1%, and 59.8%, respectively), GG1 (53.8%, 42.5%, and 38.2%, respectively), and GG2, (67.8%, 75.7%, and 73%, respectively). However, in patients with GG ≥ 3, MRI-fusion TB + SB and TB showed significantly higher concordance with prostatectomy histopathology compared to TRUS-Bx (73.1% vs. 42.9%, p = 0.018; 71.4% vs. 42.9%, p = 0.035, respectively). No significant differences were found in upgrading rates between these groups in all pathological categories. The downgrading rate was significantly lower in MRI-fusion TB + SB and TB groups compared to TRUS-Bx in all GG except GG1 (**Table 3**). **Figures 1A–C** demonstrated concordance, upgrade, and downgrade rates of prostatectomy histopathology with TRUS-Bx, MRI-fusion TB + SB, and TB groups stratified by ISUP GG.

**Table 2:** Correlation of ISUP Grade Groups obtained by different biopsy techniques with radical prostatectomy histopathology.

TRUS-Bx N=115	Radical Prostatectomy GG, n						Concordance %
	GG 1	GG 2	GG 3	GG 4	GG 5	Total	
GG 1	**28**	20	4	0	0	52	53.8
GG 2	5	**19**	3	1	0	28	67.9
GG 3	0	9	**8**	0	0	17	47.1
GG 4	0	2	6	**6**	2	16	37.5
GG 5	0	0	0	1	**1**	2	50
Total	33	50	21	8	3	**115**	
MRI-TB+SB N = 103	Radical Prostatectomy GG, n						Concordance %
	GG 1	GG 2	GG 3	GG 4	GG 5	Total	
GG 1	**17**	17	4	1	1	40	42.5
GG 2	2	**28**	4	0	3	37	75.7
GG 3	0	2	**10**	0	1	13	76.9
GG 4	0	0	1	**7**	2	10	70
GG 5	0	0	1	0	**2**	3	66.7
Total	19	47	20	8	9	**103**	
MRI-TB N = 92	GG 1	GG 2	GG 3	GG 4	GG 5	Total	
GG 1	**13**	15	4	1	1	34	38.2
GG 2	1	**27**	7	1	1	37	73
GG 3	0	2	**7**	0	2	11	63.6
GG 4	0	0	1	**6**	1	8	75
GG 5	0	0	0	0	**2**	2	100
Total	14	44	19	8	7	**92**	

Bold values: concordance biopsy histopathology with radical prostatectomy specimens.

MRI-TB + SB, MRI-fusion targeted biopsy (MRI-TB) + systematic PB; PI-RADS, Prostate Imaging Reporting and Data System score v2.1; ISUP GG, International Society of Urological Pathology (ISUP) Grade Groups (GG).

**Table 3:** Comparison of concordance, upgrading, and downgrading of radical prostatectomy histopathology between TRUS Bx, MRI-TB + SB, and MRI-TB histopathology.

Overall ISUP GG					
	TRUS-Bx, N= 115; n (%)	MRI-TB+SB, N = 103; n (%)	p-value^1^	MRI-TB, N = 92; n (%)	p-value^1^
**Concordance**	62 (53.9)	64 (62.1)	0.14	55 (59.8)	0.3
**Upgrading**	30 (26.1)	33 (32.1)	0.2	33 (35.9)	0.09
**Downgrading**	23 (20)	6 (5.8)	**0.002**	4 (4.3)	**0.0006**
ISUP GG 1					
	TRUS-Bx; N= 52, n (%)	MRI-TB+SB, N = 40; n (%)	p-value^1^	MRI-TB, N= 34; n (%)	p-value^1^
**Concordance**	28 (53.8)	17 (42.5)	0.5	13 (38.2)	0.12
**Upgrading**	24 (46.2)	23 (57.5)	0.19	21 (61.8)	0.12
ISUP GG 2					
	TRUS-Bx; N= 28, n (%)	MRI-TB+SB, N = 37; n (%)	p-value^1^	MRI-TB, N= 37; n (%)	p-value^1^
**Concordance**	19 (67.8)	28 (75.7)	0.33	27 (73)	0.43
**Upgrading**	4 (14.3)	7 (18.9)	0.44	9 (24.3)	0.24
**Downgrading**	5 (17.9)	2 (5.4)	0.12	1 (2.7)	**0.048**
ISUP GG ≥ 3					
	TRUS-Bx; N = 35; n (%)	MRI-TB+SB, N = 26; n (%)	p-value^1^	MRI-TB, N = 21; n (%)	p-value^1^
**Concordance**	15 (42.9)	19 (73.1)	**0.018**	15 (71.4)	**0.035**
**Upgrading**	2 (5.7)	2 (7.7)	0.6	3 (14.3)	0.57
**Downgrading**	18 (51.4)	5 (19.2)	**0.0001**	3 (14.3)	**0.005**

^1^Chi-squared test, Fisher’s exact test.

Bold values: statistically significant p-values (p <0.05).

MRI-TB + SB, MRI-fusion targeted biopsy (MRI-TB) + systematic prostate biopsy (SB); ISUP GG, International Society of Urological Pathology Grade Groups.

The study revealed a positive correlation between PI-RADS 3 (p = 0.002) and PI-RADS 4 suspicion score (p = 0.04) with surgical ISUP GG2 and a strong correlation between PI-RADS 5 and ISUP GG ≥ 3 (p < 0.0001) (**Table 4**).

**Table 4:** Correlation of mpMRI PI-RADS with histopathology (ISUP GG) after radical prostatectomy in MRI-fusion targeted + systematic prostate biopsy group N =103.

PI-RADS v2.1	ISUP GG 1, n (%)	ISUP GG 2, n (%)	ISUP GG ≥ 3, n (%)	p = value^1^
**PI-RADS 2,** n = 4	3 (75)	1 (25)	_	
**PI-RADS 3,** n = 25	4 (16)	15 (60)	6 (24)	**0.002**
**PI-RADS 4,** n = 48	12 (25)	22 (45.8)	14 (29.2)	**0.04**
**PI-RADS 5,** n = 26	1 (3.8)	8 (30.8)	17 (65.4)	**<0.0001**

^1^Chi-squared test.

Bold values: statistically significant p-values (p <0.05).

PI-RADS, Prostate Imaging Reporting and Data System score v2.1; ISUP GG, International Society of Urological Pathology Grade Groups.

**Tables 5A**, **B** compare the number of cancer-involved regions in patients with concordant, upgraded, and downgraded prostatectomy histopathology. **Table 5A** shows a significant statistical difference in the number of cancer-involved regions between concordant and upgraded prostatectomy specimens; however, no difference is observed with downgraded histopathology, likely due to the small patient sample.

**Table 5A:** Comparison of the number of cancer-involved regions between concordance and upgrading of prostatectomy histopathology.

	Concordance		Upgrading			
	#	Mean (SD)	#	Mean (SD)	95% CI	P value
**No. cancer-involved regions**	64	3.03 (1.347)	33	2.485 (1.034)	2.699 to 3.361	**0.0438**

Bold values: statistically significant p-values (p <0.05).

**Table 5B:** Comparison of the number of cancer-involved regions between concordance and downgrading of prostatectomy histopathology.

	Concordance		Downgrading			
	#	Mean (SD)	#	Mean (SD)	95% CI	P value
**No. cancer-involved regions**	64	3.03 (1.347)	6	2.167 (1.169)	2.118 to 2.852	0.1338

In the multivariate logistic regression model for MRI-fusion PB with TRUS Bx, the type of biopsy was a predictive factor for downgrading (OR = 2.22, 95% CI = 1.01-5.14, p = 0.05) (**Table 6A**). Two multivariate models were created to analyze predictors of histopathologic concordance, upgrading, and downgrading of prostatectomy specimens for MRI-fusion PB. In both models, the number of cancer-involved regions was a predictor for concordance (OR = 1.24, 95% CI = 1.04-1.52, p = 0.02) and downgrading (OR = 0.68, 95% CI = 0.46-0.94, p = 0.03). PI-RADS score was a predictor for upgrading (OR = 0.46, 95% CI = 0.24-0.83, p = 0.01), and MCCL was a predictor for downgrading (OR = 0.77, 95% CI = 0.6-0.95, p = 0.02) (**Table 6B**).

**Table 6:** Multivariate logistic regression analysis to identify prognostic factors for concordance, upgrading, and downgrading prostatectomy specimens compared with the histopathology of **A**. MRI-fusion TB+SB with TRUS PB (**N = 218**) and **B**. MRI-fusion TB+SB only (**N = 103**).

A.									
Variables	Concordance			Upgrading			Downgrading		
	OR	95% CI	p-value	OR	95% CI	p-value	OR	95% CI	p-value
**Clinical stage**	1.59	0.76-3.40	0.2	0.47	0.19-1.08	0.09	1.30	0.45-3.45	0.6
**PSA**	0.94	0.88-1.01	0.1	1.05	0.97-1.13	0.2	1.04	0.94-1.13	0.4
**PV**	1.01	0.99-1.02	0.4	0.99	0.98-1.01	0.3	1.00	0.98-1.02	>0.9
**MCCL**	1.07	1.0-1.15	0.07	0.96	0.89-1.04	0.3	0.94	0.84-1.03	0.2
**Type: MRI PB vs. TRUS-Bx**	0.99	0.56-1.74	>0.9	0.62	0.33-1.14	0.12	2.22	1.01-5.14	**0.05**
B. Model 1									
Variables	Concordance			Upgrading			Downgrading		
	OR	95% CI	p-value	OR	95% CI	p-value	OR	95% CI	p-value
**MCCL**	1.02	0.92-1.13	0.7	1.06	0.96-1.19	0.3	0.77	0.6-0.95	**0.02**
**PI-RADS score**	1.48	0.87-2.58	0.2	0.5	0.27-0.89	**0.02**	2.12	0.79-6.49	0.2
**No. Cancer-involved regions**	1.24	1.03-1.5	**0.03**	0.9	0.74-1.09	0.3	0.69	0.46-0.95	**0.04**
B. Model 2									
Variables	Concordance			Upgrading			Downgrading		
	OR	95% CI	p-value	OR	95% CI	p-value	OR	95% CI	p-value
**PSA**	0.94	0.84-1.04	0.2	1.1	0.98-1.24	0.11	0.95	0.78-1.12	0.6
**PI-RADS score**	1.69	0.97-3.04	0.07	0.46	0.24-0.83	**0.01**	1.75	0.68-4.92	0.3
**No. Cancer-involved regions**	1.24	1.04-1.52	**0.02**	0.91	0.74-1.1	0.3	0.68	0.46-0.94	**0.03**

OR, Odds Ratio; CI; Confidence Interval. Bold values: statistically significant p-values (p <0.05).

MRI-fusion TB + SB = MRI-fusion targeted + systematic prostate biopsy; PI-RADS, Prostate Imaging Reporting and Data System score v2.1; ISUP GG, International Society of Urological Pathology Grade Groups; MCCL, Maximum cancer core length; PV, prostate volume.

## Discussion

Early published studies on overestimation and underestimation of histopathology after prostatectomy showed that MRI-fusion TB is superior to TRUS-Bx in correctly diagnosing GS of PCа (13, 15). In 2016, Porpiglia et al. found that TB has better concordance with prostatectomy specimens than TRUS-Bx (91.5% vs. 53.8%, p <0.001). In their study, TB also reduced the risk of GS upgrade (7.8% vs. 39.3%, p <0.001) and downgrade (0.8% vs. 6.8%, p <0.001) compared to TRUS-Bx in all grade categories (16). In a more recent study, Diamand et al. found no significant difference in concordance with prostatectomy histopathology between TRUS-Bx and MRI-fusion TB groups (49.4% vs. 51.2%) for overall ISUP GG; however, for ISUP GG ≥ 2, the concordance, upgrading, and downgrading rates were significantly better in the MRI-TB group than in the TRUS group (p < 0.001) (14). Luzzago et al. also reported improved concordance with prostatectomy histopathology in MRI-fusion TB compared to TRUS Bx in patients with ISUP GG2 (71 vs. 54.9%; p = 0.04) and GG ≥ 3 (65 vs. 39%; p < 0.01) (25). The findings from our investigation are consistent with the results of recent studies.

We found no significant difference in the concordance and upgrading of prostatectomy histopathology between the MRI-fusion PB and TRUS-Bx groups for the overall ISUP GG. However, in patients with unfavorable intermediate and high-risk PCa (ISUP G ≥ 3), the concordance of prostatectomy histopathology with MRI-fusion PB was significantly better than with the TRUS-Bx.

Some studies have shown that combining MRI-fusion TB with SB increased concordance and reduced upgrading of prostatectomy specimens (14, 17). Our investigation did not find that combining MRI-fusion TB with SB improved the concordance and reduced downgrading and upgrading of prostatectomy histopathology compared to TB alone in all ISUP GG.

Until recently, the criteria for selecting patients for active surveillance (AS) included GS, clinical stage, PSA, PSA density, number of positive biopsy cores, and MCCL. Based on 12-cores TRUS Bx results, patients meeting the Epstein or D’Amico criteria for low- and favorable intermediate-risk PCa were eligible for AS (26). With the addition of mpMRI in the diagnostic pathway for PCa, some uncertainty remains regarding the selection criteria for AS based on different MRI-fusion PB protocols. There is currently no consensus on the optimal schemes for SB sampling after MRI-fusion TB. Frequently used in clinical practice, the Ginsburg biopsy technique for transperineal MRI-fusion PB requires 2 to 4 TB cores, followed by 24 SB cores consisting of 4 cores in 6 sectors: the posterior, mid-gland, and anterior bilaterally (27). We use the MRI-fusion PB scheme, which includes 2 to 4 TB and 20 SB cores collected from 5 prostate regions (24). In the transrectal approach, after TB, 12 cores of SB are usually taken from 6 prostate regions: apex, mid-gland, and base bilaterally (12–14). Although the number of SB cores taken in various MRI-fusion PB protocols can differ significantly, the number of regions of the prostate for SB is generally limited to 6. In classical 12-cores TRUS-Bx, the number of positive biopsy cores was often used as a variable in multivariate analysis to assess the risk of grade reclassification in patients with AS or the histopathological concordance of prostatectomy specimens (14, 25, 28). With a different number of SB cores in the various protocols of MRI-fusion PB, this variable cannot be used. When using MRI-fusion PB to diagnose PCa, we propose that the criteria for patient selection for AS or focal therapy should be based on the number of cancer-involved regions rather than the number of positive biopsy cores. Standardizing this variable is straightforward. This criterion can also define the multifocality of the disease, which is essential when selecting patients for focal treatment of prostate cancer.

Therefore, we replaced the number of positive biopsy cores with the number of cancer-involved regions as a variable in the multivariate analysis, and this criterion was an independent predictor of pathological concordance. А comparison of the number of cancer-involved regions in patients with concordant, upgraded, and downgraded prostatectomy histopathology showed a significant statistical difference between concordant and downgraded prostatectomy histopathology; however, no difference was observed in upgraded histopathology.

To the best of our knowledge, the number of cancer-involved regions has not been applied in the literature in terms of decision-making regarding active surveillance and other treatments.

А recent systematic review and meta-analysis do not recommend using serial mpMRI as a criterion for excluding PCa progression in AS patients (29). Hsiang et al. found no association between mpMRI progression and pathological upgrade. However, a PI-RADS score of 4-5 on mpMRI predicted subsequent pathological progression (30). Luzzago et al. concluded that AS should be discouraged in patients with PI-RADS 5 lesions in the initial biopsy due to the high likelihood of histopathology upgrading at prostatectomy (25). Our findings are consistent with these studies. We observed a direct correlation between higher PI-RADS scores and higher ISUP GG after prostatectomy. Specifically, PI-RADS 3 (p = 0.002) and PI-RADS 4 (p = 0.04) correlated with surgical ISUP GG2, whereas PI-RADS 5 is associated with ISUP GG ≥ 3 (p < 0.0001). Additionally, in the multivariable analysis, the PI-RADS score was an independent predictor for upgrading prostatectomy histopathology. Based on our investigation, we propose that PI-RADS 5 on initial mpMRI should be an exclusion criterion for AS of PCa patients.

In our study, MRI-fusion PB demonstrated significantly less histopathology downgrading at prostatectomy than TRUS-Bx in all grade categories. According to the multivariable analysis, using the MRI-fusion PB approach is an independent predictor for reducing the downgrading of prostatectomy histopathology. There are conflicting reports about the predictive value of GS upgrading or downgrading at prostatectomy and its impact on worse outcomes. Tilki et al. reported that patients with upgraded prostatectomy histopathology were more likely to have an extracapsular extension, seminal vesicle invasion, positive surgical margins, and lymph node involvement at prostatectomy (31). Other studies investigating the influence of adverse prostatectomy histopathology found that upgraded histopathology does not significantly affect oncological outcomes or mortality (32, 33). On the contrary, downgrading the PCa diagnosis at prostatectomy suggests that the patient received unnecessary treatment or that the surgery could have been avoided (33, 34). Recently, Wang et al., in a study of 99,835 PCa patients who underwent prostatectomy between 2010 and 2017, found that 18.5% had a histopathology downgrading, resulting in a 45% increased risk of cancer-related mortality compared with patients without a downgrading for any grade categories (35).

The advancement of mpMRI software-based fusion techniques improves cancer detection and characterization, and it is reasonable to expect that this will significantly improve the accuracy of GS diagnosis. However, the GS has been accurately diagnosed only in two-thirds of cases. Various groups of Gleason grade included in ISUP GG4 and ISUP GG5 PCa may lead to incorrect identification of a dominant pattern (36, 37). Additionally, mpMRI has a high sensitivity for detecting csPCa and very low sensitivity for ciPCa, leading to selective sampling from areas of higher grade seen on mpMRI in low-grade PCa (38). Another factor that creates discordance is the multifocality and heterogeneity of disease found in 87% of prostatectomy specimens. The majority of separate tumors in the same prostate specimen had different Gleason grades, and a small number of foci of high-grade PCa were identified in specimens with low-grade PCa (39).

Limited interobserver reproducibility among pathologists may cause inaccurate assessment of the ISUP GG (16, 40). Diamand et al. suggests incorrect ISUP grading may be due to limited interobserver reproducibility among pathologists. The pathologists still disagree on assigning Gleason Scores and ISUP grades. In his opinion, this problem would be solved if the same pathologist analyzed the biopsy and the final prostatectomy specimen (14).

Our study has limitations. First, the analysis was retrospective, which makes it more prone to bias. Second, we compared different cohorts of patients undergoing MRI-fusion PB and TRUS-Bx. However, we ensured the inclusion of matched patient groups in the study. It may be methodologically incorrect to compare TB and SB results in the same patient. However, the study design reflects our aim to evaluate the improvement in concordance of prostatectomy histopathology with a new approach, the transperineal MRI fusion PB, compared to classical 12-cores TRUS-Bx. According to current recommendations, we performed an MRI-fusion TB in combination with an SB. Therefore, it is difficult to accurately measure the potential benefit of TB alone at the time of combined biopsy.

An explanation is needed for the high percentage of patients undergoing radical prostatectomy with an ISUP GG1 in group 1. Our two patient groups demonstrate the historical shift in AS criteria from the Epstein and D’Amico recommendations to the most recent EAU guidelines. In the first group, we applied the strict inclusion criteria for the AS that were accepted at that time. Patients who did not meet these criteria were offered radical prostatectomy, even if they had ISUP GG1 PCa. Thus, in group 1, 45.2% of patients diagnosed with ISUP GG1 PCa were referred for surgery, and 38.9% were confirmed to have postoperative ISUP GG1 PCa. We revised the inclusion criteria for the AC protocol following the publication of many studies providing compelling evidence to support the inclusion of ISUP GG2 prostate cancer patients in the AC protocol. This shift is evident in group 2, where only 19.4% of patients were diagnosed with postoperative ISUP GG1 PCa.

Finally, our investigation is based on analyzing biopsy outcomes from a single tertiary center with a limited patient sample size, which may result in a lack of statistical significance.

## Conclusion

In ISUP GG1 and GG2 PCa, we found no significant differences between the biopsy groups in concordance and upgrading of prostatectomy histopathology. For ISUP GG ≥ 3 PCa, MRI-fusion PB significantly improved concordance compared with TRUS-Bx. Additionally, MRI-fusion PB reduces the overestimation of disease at prostatectomy for all grade categories.

With the widespread use of MRI-fusion prostate biopsy for diagnosing prostate cancer, new criteria related to biopsy results are necessary to assess the disease’s potential for adverse outcomes. Our study found that the number of cancer-involved regions is an independent predictor of the concordance between biopsy and prostatectomy histopathology and the overestimating disease on biopsy. This criterion should be considered when selecting patients for AS or focal therapy instead of the number of positive cores. Prospective randomized trials are necessary to validate our retrospective, single-institution findings and their significance in clinical decision-making for patients with PCa, based on the histopathology obtained from MRI-fusion PB.

## Data Availability

All relevant data are within the manuscript. However, according to National laws and regulations, the data cannot be uploaded to the repository. The data can be shared upon request and addressed to Prof. Eitan Lunenfeld, MD, MHA, Head of I.R.B., Soroka University Medical Center, Be'er-Sheva, Israel. eitan_l@clalit.org.il.
